# Algorithm for the Comparison of Human Periodic Movements Using Wearable Devices

**DOI:** 10.1155/2021/8729108

**Published:** 2021-12-09

**Authors:** Marlon Burbano-Fernandez, Jhoana Sandoval-Serna, Yilton Riascos, Mario Muñoz-Organero, M. Thilagaraj, V. Venkataraman, N. Arunkumar, Gustavo Ramirez-Gonzalez

**Affiliations:** ^1^Departamento de Telemática, Universidad del Cauca, Popayán, Colombia; ^2^Facultad de Ciencias Contables, Económicas y Administrativas, Universidad del Cauca, Popayán, Colombia; ^3^Departamento de Matemáticas, Universidad del Cauca, Popayán, Colombia; ^4^Departamento de Ingeniería Telemática, Universidad Carlos III de Madrid, Getafe, Spain; ^5^Karpagam College of Engineering, Coimbatore, India; ^6^Department of Mathematics, School of Arts Science and Humanities, SASTRA Deemed University, Thanjavur, India; ^7^Biomedical Engineering Department, Rathinam College of Engineering, Coimbatore, India

## Abstract

In the context of teaching-learning of motor skills in a virtual environment, videos are generally used. The person who wants to learn a certain movement watches a video and tries to perform the activity. In this sense, feedback is rarely thought of. This article proposes an algorithm in which two periodic movements are compared, the one carried out by an expert and the one carried out by the person who is learning, in order to determine how closely these two movements are performed and to provide feedback from them. The algorithm starts from the capture of data through a wearable device that yields data from an accelerometer; in this case, the data of the expert and the data of the person who is learning are captured in a dataset of salsa dance steps. Adjustments are made to the data in terms of Pearson iterations, synchronization, filtering, and normalization, and DTW, linear regression, and error analysis are used to make the corresponding comparison of the two datasets. With the above, it is possible to determine if the cycles of the two signals coincide and how closely the learner's movements resemble those of the expert.

## 1. Introduction

### 1.1. Background

The study of movements and the learning of motor skills is very striking. In this area, we can find studies and commercial products that show devices for the accompaniment or monitoring of physical activities. These provide relevant and essential information for different users whose objective is to learn, monitor, or record the motor activities they carry out.

In the case of learning through electronic means, in general, there is a video in which specific actions using the human body are carried out and then a person tries to imitate them (sports courses, motor skills, dance, or others). In the face-to-face context, it is observed that the apprentice sees the movements carried out by an expert and then tries to carry them out while being watched by an instructor who determines whether the skills are being carried out correctly or incorrectly and then provides recommendations on how to perform them. In this sense, it is possible to think of a system that helps to provide feedback on movements based on a previously proposed architecture [1].

Taking a look at Biomechanics, it is found that one of its many classifications is framed in the periodicity of movements, where there are activities that can be classified as periodic movements, which are characterized by being those motor skills in which the same movement is repeated several times, for example, walking, running, swimming, cycling, or dancing. This characteristic makes them of particular interest and very attractive in the research field, since this involves thinking about the possibility of capturing a pattern that arises based on monitored data of the human body and, based on the data collected, comparing patterns that allow feedback to learn the movements.

In this context, some studies use wearable devices in order to capture movements for analysis [2–5], while others aim at making some feedback towards people [6–9]. These works are framed in algorithms, generally machine learning for the classification and identification of movements. In this work, the idea is to use algorithms that do not require data training to make the recommendations.

On the other hand, in this work, DTW (Dynamic Time Warping) [10, 11] is used as the basis for making a comparison from a dataset. In works found on this subject [12–15], it is observed that the use of this algorithm implies the analysis of signals to compare them and they can be the starting point for the development of the algorithm proposed in this work, which, unlike those found, involves the comparison of periodic movements captured with wearable devices.

This article shows how to construct an algorithm for comparing periodic movements using Pearson iterations, DTW, linear regression, and error analysis. We use a dataset of salsa dance steps as samples for a study case.

### 1.2. Conceptual Framework of the Algorithm

In order to find a way in which it is possible to make a comparison between two people who perform the same motor skill, with one person acting as the expert on the movements and the other person trying to replicate this movement, the measurements of an accelerometer are selected as the data that concentrates on the information of the movements that are carried out. The data obtained, by its own nature, have a time series form. In the case of the acceleration data in its grossest form, we have the time interval *t* and the acceleration data in the *x*-, *y*-, and *z*-axes in measures of the force of gravity *g* or meters per second m/s^2^.

However, to facilitate the analysis, the data are converted into a univariate series using the modulus of the acceleration components. The modulus is calculated under the formula $v=\sqrt{x^{2}+y^{2}+z^{2}}$. The main reason for using the acceleration module is that the data in the different axes can vary according to the position of the device on the person. The vertical axis of the accelerometer, in an ideal case, should coincide exactly with the alignment of the Earth's attraction. However, it is complicated to apply this parameter in a poorly controlled context, since not all people will carry out this alignment. To solve this problem, there are two possibilities: The first possibility is to adjust a mathematical way in the axes, where a zero time in which there are no movements is considered, so that the compensation is made in a calculated way in the axes. The second possibility is to convert the multivariate series to univariate based on the use of the module. In this case, the second option was chosen.

With the adjusted data, we must find a way to compare them; in this case, the problem is to find out how two time series compare. Graphically, it is easily detectable with the naked eye if their behaviors are similar, which now conveys the problem in how similar this pair of signals can become, involving various factors to overcome. One of them is that, due to Newton's second law, the acceleration varies inversely depending on the mass (Force=mass*∗*acceleration). This can be seen reflected in the fact that two people of different weight and height can perform the same movement, but the reading of their data can be different. Another factor that may arise is that it is essential to control the temporal parameter, since when performing periodic movements (dance steps, walking, running, swimming, or pedaling), the number of samples obtained per unit of time must be controlled. In this case, two situations can arise; the first is that the movement is independent of the frequency that is needed; for example, two samples can be taken from a person who performs 60 steps per minute and from another person who performs 30 steps in a minute. Or, in the case of swimming, that one person performs 50 strokes, while another person performs 40 in the same time period, time, without this being relevant to the imitation of the movement. However, there are other cases where the frequency of the movement does represent relevance, for example, in the case of dancing, where a temporary measure is required to indicate when a movement is performed correctly. Usually, this is done according to the beat of the music or with the use of metronomes. Finally, a third parameter to be considered is performing the movement itself in the correct way, where the data thrown must determine if the movements are made in a similar way from the data.

Now, when dealing with time series, it is possible to think about using their characteristics for their study. The time series have the components of seasonality (pattern of change, regularly recurring over time), trend (determines growth or decline), cyclicality (fluctuations in waveform or cycles), and randomness (irregular behavior is composed of fluctuations caused by unpredictable or nonrecurring events). It can be thought that, from these characteristics, it is possible to determine important values for comparison; for example, randomness and trend are related to the actual performance of the movement, and cyclicality and seasonality are reflected in the temporal and seasonal characteristics—repetition of movement.

From the functional point of view, the users, regardless of whether they are expert users or the ones performing the movement by imitation, need a system of recommendations which tells them if the compared movements are the same or different and, based on this, they receive a particular type of refeeding, as a recommendation for a subsequent attempt. On the other hand, one can also think of other types of applications that can be taken into account to use the algorithm, for example, while comparing the movements performed by a person recovering from a sports injury, recorded at different moments, comparing the movements of a person who uses some prosthesis and those of an average person, and detecting through the comparison of the movement's shortcomings in athletes' performances or finding optimal movements.

## 2. Materials and Methods

### 2.1. General Parameters

**Figure 1:**
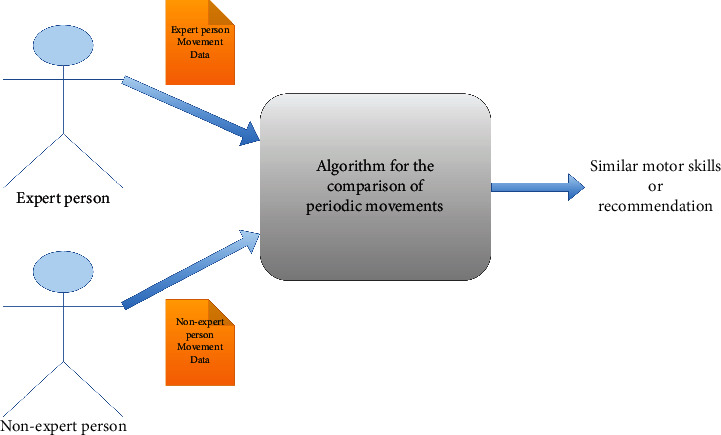
Black box of the algorithm for the comparison of periodic movements.

Seeing it as a black box in Figure 1, the algorithm for the comparison of movements would receive two univariate time series. According to the characteristics of the time series, it will carry out processes that indicate whether the movements are similar and then provide a recommendation to achieve the similarity of movements later.

### 2.2. Initial Approach of the Algorithm

**Figure 2:**
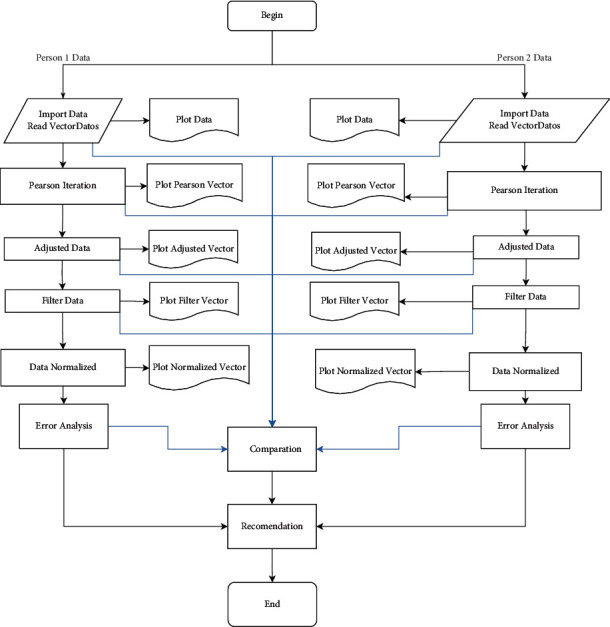
General parameters of the algorithm.

A general structure is then proposed for the movement comparison algorithm represented in Figure 2. The image shows that the proposed algorithm behaves in a linear and parallel way based on the data entered by two people who want to compare some periodic movements. As mentioned previously, the data to be entered is required to be found in the first measure, collected with the same sampling rate and in the same unit of measure, *g* or m/s^2^.

In general, each module that makes up the algorithm is responsible for linearly adjusting the original data; that is, the data enters each module; after some processing, it delivers the data required to display them graphically and perform comparative analysis for each module.

The ImportData module reads the data in the algorithm and places it in vector format, *w*=[*a*_1_; *a*_2_; *a*_3_; …; *a*_*n*_], where *a*_*i*_ corresponds to the accelerometer reading at each instant.

The AjustedData module adjusts so that the vectors generated in the ImportData module start similarly and are adjusted in the same phase. From this module, the data vectors will be considered signals due to their representation from a Cartesian plane. The PearsonIteration module is in charge of determining the periodicity of the signals and comparing them in terms of the period focused on each one.

The FilterData module smoothes the signals in order to eliminate noise that may be present in the data. The DataNorrmalized module performs a signal scale adjustment to facilitate data comparison. The error analysis module determines the proximity between one signal and the other in order to determine their degree of similarity from linear regression analysis and its error. The Comparison module performs the comparison of the signals that are generated throughout the algorithm. Finally, the Recommendation module delivers a result of the algorithm translated into a recommendation to be followed, so that the movements have a high degree of similarity or recognize their similarity.

### 2.3. Data Import

The data collected is obtained from wearable devices or an application that uses sensors to capture information on the acceleration of people while making movements. The data must be captured in a univariate time series, where the acceleration values through time are used. In this case, the magnitude of the acceleration for all moments is taken as the value to be entered into the algorithm, and it is represented under the nomenclature *x*_*t*_.

Since the acceleration measurements can vary according to the nomenclature used, in either gravities *g* or meters per second squared m/s^2^, it must be ensured that the measurements that enter the algorithm are in the same units. On the other hand, it must be taken into account that the frequency of the capture by the wearable or the application corresponds to the devices used by the two people who carry out the capture; that is, if the capture of 20 pieces of data per second is made for one person *x*, the other person *y*, who wants to compare the movement, must also consider a total of 20 pieces of data per second.

**Figure 3:**
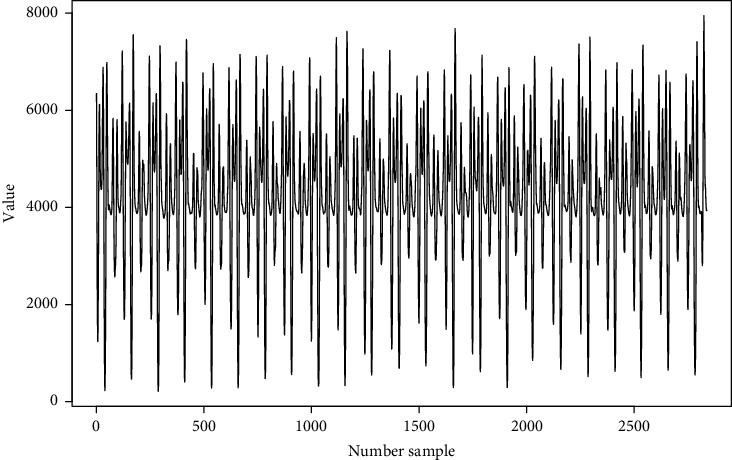
Time series example.

From the acceleration data collected with a wearable device, a sample is selected as an example of the data obtained and represented in the form of a signal from a wearable device (in this case, the data is in https://github.com/mfburbano/SalsaDanceDataSet/blob/main/User2SetepForwardBackward/Sample_1/AnalysisData_raw_0.csv), where on the vertical axis we have the magnitude of the acceleration, in this case (m/s^2^*∗*100), and, on the horizontal axis, the samples taken over time, as shown in Figure 3.

### 2.4. Pearson Iterations

**Figure 4:**
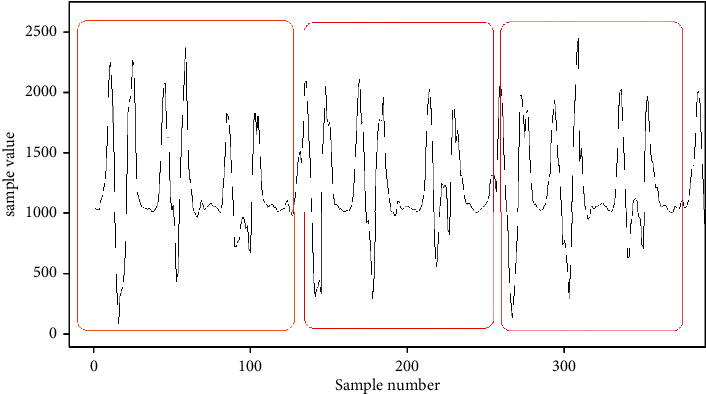
Example of enlarged graphical representation of samples.

Correlations are defined as the correspondence between two signals; in Pearson's coefficient, the values are between −1 and 1, where 1 is a perfect positive linear correlation and −1 is a perfect negative linear correlation. Under this principle, if two signals that contain the same number of samples are equal, their Pearson correlation will be 1, or if they are similar, their value will be close to 1. Let us remember that, when evaluating periodic movements, the expected logical response is that the periodicity is reflected in the signals. If we go back to the example given in Figure 3, on a larger scale (Figure 4), with the naked eye, it can be seen that there is a repetition of a pattern. Under the two previous premises, we can think about Pearson's coefficient to determine this cyclicality. To do this, we define the term *Pearson iterations*, where a signal is iterated on itself to determine its cyclicity. Let us suppose the signal that represents a periodic behavior *v*, composed of the data *x*_1_; *x*_2_; *x*_3_; *x*_4_; …; *x*_*n*_; that is, we can define the vector:

(1) $$\begin{array}{c} v=\left[x_{1},x_{2},x_{3},x_{4},\ldots ,x_{n}\right]. \end{array}$$

From vector *v*, we can shift the signal data in a carousel fashion and define the vectors:

(2) $$\begin{array}{c} v_{1}=\left[x_{n},x_{1},x_{2},x_{3},\ldots ,x_{n-1}\right]\\ v_{2}=\left[x_{n-1},x_{n},x_{1},x_{2},\ldots ,x_{n-2}\right]\\ v_{3}=\left[x_{n-2},x_{n-1},x_{n},x_{1},\ldots ,x_{n-3}\right]\\ \vdots \\ v_{n+1}=\left[x_{n},x_{1},x_{2},x_{3},\ldots ,x_{n-1}\right]. \end{array}$$

We define the Pearson correlation of vector *v* and vector *v*_*n*_ as Pearson_*v*,*v*_*n*__, where Pearson_*v*,*v*_*n*__=1, if vector *v* = vector *v*_*n*_ so that Pearson_*v*,*v*_*n*__=1 or if vector *v* ≠ vector *v*_*n*_, Pearson_*v*,*v*_*n*__ ≠ 1.

Now, we can perform Pearson correlations between vector *v* and vectors *v*_1_, *v*_2_, *v*_3_, *v*_4_,…, *v*_*n*_, namely,

(3) $$\begin{array}{c} P_{1}={\text{Pearson}}_{v,v_{1}}P_{2}={\text{Pearson}}_{v,v_{2}}P_{3}={\text{Pearson}}_{v,v_{3}}P_{4}={\text{Pearson}}_{v,v_{4}}\ldots P_{n}={\text{Pearson}}_{v,v_{n}}. \end{array}$$

From this, we can create vector *P*=[*P*_1_, *P*_2_, *P*_3_, *P*_4_,…, *P*_*n*_], which corresponds to the vector of textit Pearson iterations.

As they are cyclical signals, it is expected that each time a cycle is fulfilled, a maximum value *P*_max_ between −1 and 1 can be observed. As the signal slides to do the following iteration, the value can decrease and then rise to obtain a maximum value again, so that if we know the number of iterations that occur between maximum and maximum, we can establish the period of the time series from the data.

Let us return to the example of Figure 4, where, from the data captured by an accelerometer, we can perform the previously defined operation with Pearson's iterations.

**Figure 5:**
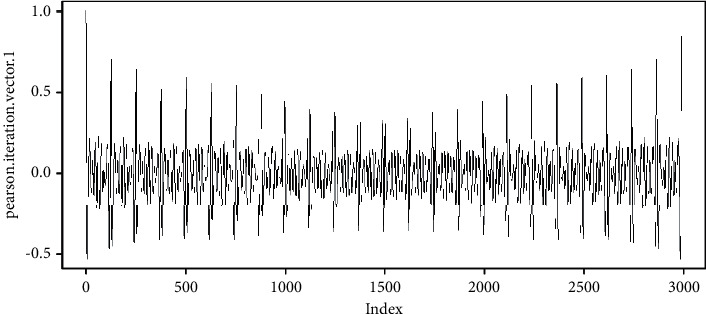
Example results of Pearson's iteration.

In this case, *v* contains 2991 samples (Figure 3), and the result of Pearson's iterations is shown in Figure 5, and the data is collected in https://github.com/mfburbano/PearsonResult/blob/main/User2StepForwardBackward/PearsonSample1.csv.

**Figure 6:**
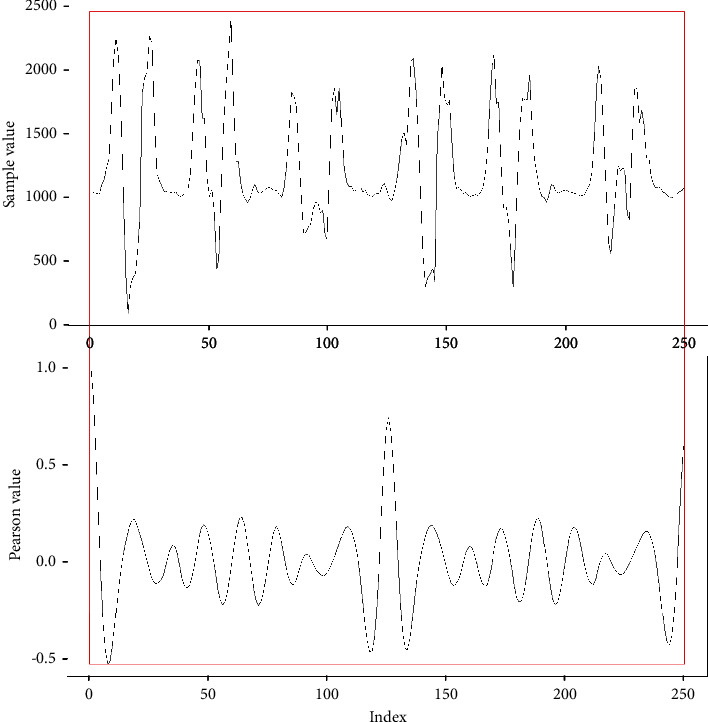
Example results of Pearson's iteration against data.

If the image is enlarged and the samples are contrasted against the result of the Pearson iterations, it can be seen that, in this case, every 125 iterations have a maximum value, with which we can conclude that every 124 samples have a period. As shown in Figure 6, in the upper part of the graph, the sampled signal is observed, and, in the lower part, the graph from Pearson's iterations and the periods is framed in red rectangles.

### 2.5. Data Adjustment

Let us remember that the algorithm must receive two sets of data. As they are different signals, it is necessary to determine if the beginnings of the signal correspond in both cases; for this, a data adjustment is made to guarantee that the signals start with similar behaviors. From two actions, it is sought that, first, the two signals start with similar behaviors and that the amount of data is the same for the two samples.

In this order of ideas, we take our vector *v*=[*x*_1_, *x*_2_, *x*_3_, *x*_4_,…, *x*_*n*_] as our first sample and *u*=[*y*_1_, *y*_2_, *y*_3_, *y*_4_,…, *y*_*m*_] as the second set of data and the sample to be compared and that has a periodic behavior similar to the data of vector *v*.

Taking into account the fact that the period has previously been determined from the Pearson iterations and that, in turn, they contain the same period, the general idea is to start from the first maximum of the first cycle for both samples and then cut the number of samples in which it has the smallest number so that the comparison can be made with the same amount of data.

For the samples to start at the first maximum, we take vectors *v*′=[*x*_1_, *x*_2_, *x*_3_, *x*_4_,…*x*_*n*′_] and *u*′=[*y*_1_, *y*_2_, *y*_3_, *y*_4_,…, *y*_*m*′_], where *n*′=*m*′ and *y* corresponds to the number of samples for a cycle.

Using the Pearson iterations, we can do the Pearson correlation, moving one vector and fixing the second; that is, we define a vector *R* as the iterations of Pearson between the data of the first cycle between vectors *v*′ and *u*′ so that, in this case, vector *v*′ is fixed and vector *u*′ is shifted:

(4) $$\begin{array}{c} v^{\prime }=\left[x_{1},x_{2},x_{3},x_{4},\ldots x_{n^{\prime }}\right],\\ u^{\prime }=\left[y_{1},y_{2},y_{3},y_{4},\ldots ,y_{n^{\prime }}\right],\\ u_{1}^{\prime }=\left[y_{n^{\prime }},y_{1},y_{2},y_{3},\ldots ,y_{n^{\prime }-1}\right],\\ u_{2}^{\prime }=\left[y_{n^{\prime }-1},y_{n^{\prime }},y_{1},y_{2},\ldots ,y_{n^{\prime }-2}\right],\\ \vdots \end{array}$$

(5) $$\begin{array}{c} R_{0}={\text{Pearson}}_{v^{\prime },u^{\prime }}\\ R_{1}={\text{Pearson}}_{v^{\prime },u_{1}^{\prime }}\\ R_{2}={\text{Pearson}}_{v^{\prime },u_{2}^{\prime }}\\ R_{3}={\text{Pearson}}_{v^{\prime },u_{3}^{\prime }}.... \end{array}$$

So *R*=[*R*_1_, *R*_2_, *R*_3_, *R*_4_,…, Racs_*n*′_].

We define the max() function as the one that determines the maximum value in a dataset and its position, so that max(*R*)=(*R*_max_, *P*_*R*_max__), where *R*_max_ is the maximum value reached in the dataset and *P*_*R*_max__ corresponds to the position where the maximum value is found. Once this position is known, it is possible to slide the data of vector *u* up to a vector called *u*_adj_, so that they coincide with the highest Pearson iteration in such a way that the coincidence in the data is higher; that is,

(6) $$\begin{array}{c} v=\left[x_{1},x_{2},x_{3},x_{4},\ldots ,x_{n}\right], \end{array}$$

(7) $$\begin{array}{c} u_{\text{adj}}=\left[y_{P_{R_{\max}}},y_{\left(P_{R_{\max}}+1\right)},y_{\left(P_{R_{\max}}+2\right)},y_{\left(P_{R_{\max}}+3\right)},\ldots y_{\left(m-P_{R_{\max}}\right)}\right]. \end{array}$$

Once this is done, it must be ensured that the amounts of data match for the two sets. In this case, it must be determined which one is the dataset with the smallest amount of data, that is, determining if *n* < (*m* − *P*_*R*_max__) or (*m* − *P*_*R*_max__) < *n*. In this case, let us call *p*=*n* if *n* is the smallest value or *p*=(*m* − *P*_*R*_max__) in case *P*_*R*_max__ is the lower value, so that we discard the data found after position *p*, in either set *v* or *u*_*a*  *dj*_, guaranteeing that the two datasets contain the same quantity. That is, the adjusted data correspond to *v*=[*x*_1_, *x*_2_, *x*_3_, *x*_4_,…, *x*_*p*_] and

(8) $$\begin{array}{c} u_{\text{adj}}=\left[y_{P_{R_{\max}}},y_{\left(P_{R_{\max}}+1\right)},y_{\left(P_{R_{\max}}+2\right)},y_{\left(P_{R_{\max}}+3\right)},\ldots y_{p}\right]. \end{array}$$

**Figure 7:**
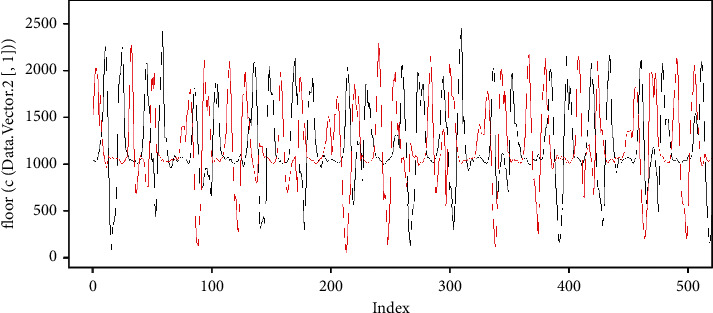
Time series example, in black *v* and in red *u*.

We return to the example, where we assign data to *v*. Now we define vector *u* (the data is in https://github.com/mfburbano/SalsaDanceDataSet/blob/main/User3SetepForwardBackward/Sample_1/AnalysisData_raw_0.csv), which visually has a behavior similar to *v* as shown in Figure 7.

As mentioned in the procedure, we only take the first cycle for both vectors, where the cycles are repeated every 125 pieces of data, forming vectors *v*′ and *u*′.

**Figure 8:**
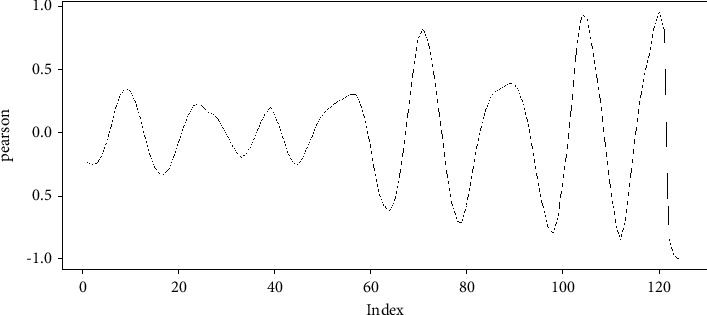
Pearson's iterations for vectors *v*′ and *u*′.

**Figure 9:**
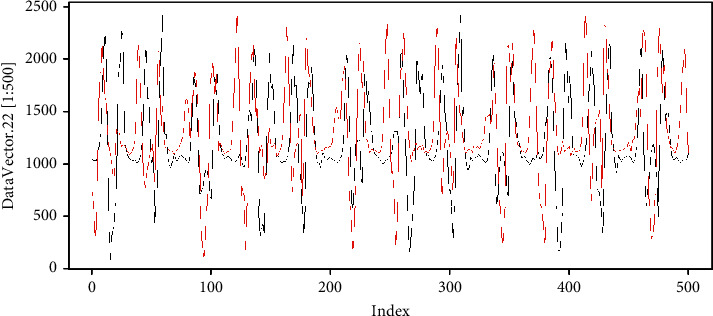
Vectors *v* and *u*_adj_.

In this case, the process mentioned in this section is carried out and results in vector *R*, which is summarized in Figure 8 that shows that the maximum value found for the Pearson iterations is given in position 120, in such a way that vector *u* is displaced; the 120 positions form vector *u*_adj_ in such a way that both vectors coincide in their cycles. In this case, the vectors are seen in Figure 9, which shows that the samples are more coincident compared to Figure 7.

### 2.6. Data Filtering

In order to minimize the noise that occurs in the signals, that is, to smooth the signals, we define an average filter with five degrees off. The filter uses a sliding window that loops through the vector data taking five consecutive elements and calculates their mean to define each element of a new vector. In this case, we take vector *v*=[*x*_1_, *x*_2_, *x*_3_, *x*_4_, *x*_5_, *x*_6_, *x*_7_, *x*_8_, *x*_9_, *x*_10_,…, *x*_*n*_], in such a way that we find the averages of every 5 elements from a scale that loops through the vector and defines each new element of the filtering vector. This is shown below:

(9) $$\begin{array}{c} v=\left[x_{1},x_{2},x_{3},x_{4},x_{5},x_{6},x_{7},x_{8},x_{9},x_{10},\ldots ,x_{n}\right]. \end{array}$$

From vector *v*, the terms of the filtered vector are defined:

(10) $$\begin{array}{c} x_{f1}=\frac{\left(x_{1}+x_{2}+x_{3}+x_{4}+x_{5}\right)}{5},\\ x_{f2}=\frac{\left(x_{2}+x_{3}+x_{4}+x_{5}+x_{6}\right)}{5},\\ x_{f3}=\frac{\left(x_{3}+x_{4}+x_{5}+x_{6}+x_{7}\right)}{5},\\ x_{f4}=\frac{\left(x_{4}+x_{5}+x_{6}+x_{6}+x_{8}\right)}{5}\\ x_{f5}=\frac{\left(x_{5}+x_{6}+x_{7}+x_{8}+x_{9}\right)}{5},\\ \vdots \\ x_{f\left(n-5\right)}=\frac{\left(x_{n-4}+x_{n-3}+x_{n-2}+x_{n-1}+x_{n}\right)}{5}. \end{array}$$

The filtered vector is

(11) $$\begin{array}{c} v_{f}=\left[x_{f1},x_{f2},x_{f3},x_{f4},x_{f5},\ldots ,x_{f\left(n-5\right)}\right]. \end{array}$$

**Figure 10:**
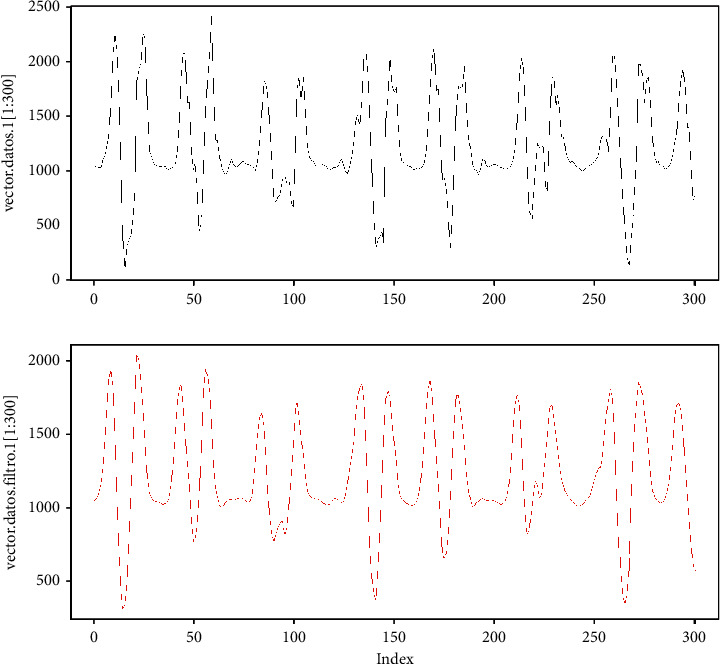
Vector filter *v*.

Returning to the example, for vector *v*, we apply the filter by average, and the result can be seen in Figure 10. In this case, it is observed that the *v*_*f*_ signal, which is in the lower part of the image, has a smoother behavior compared to the *v* signal, which is in the upper part of the figure. Also, in comparison, the number of elements of vector *v* is 2989, and the number of elements of vector *v*_*f*_ is 2985, where the last four elements are lost due to filtering. However, in this case, it is only equivalent to 0.1338% of data, and, due to the volume of the data, it does not affect the behavior of analyzed signal.

### 2.7. Normalization

Since we have two data sources as input to the algorithm, one for the samples captured by an expert and another for the samples given by a nonexpert, there may be differences in the values of the samples due to the fact that force may vary or due to the size of the people that may be different. In order to guarantee that the comparison made by the algorithm was not affected by what is described, normalization is used. For this case, it consists of the scale of the two data samples being the same.

In this case, for vector *v*, we define *x*_*n*_max__ as the sample with the highest value and *x*_*n*_min__ as the sample with the minimum value. We define *v*_norm_ as the vector where its elements are given under the formula

(12) $$\begin{array}{c} x_{i_{\text{nor}}}=\frac{x_{i}-x_{\min}}{x_{m}\text{ax}-x_{m}\text{in}}. \end{array}$$

Then,

(13) $$\begin{array}{c} v_{\text{norm}}=\left[x_{1_{\text{norm}}},x_{2_{\text{norm}}},x_{3_{\text{norm}}},\ldots ,x_{n_{\text{norm}}}\right]. \end{array}$$

Now, the normalization guarantees that the values are between −1 and 1. To make the scale more extensive, we readjust the formula and adjust it to values between −10000 and 10000, by multiplying it by 10000, so that each sample normalized will be given by

(14) $$\begin{array}{c} x_{i_{\text{nor}}}=10000*\frac{x_{i}-x_{\min}}{x_{m}\text{ax}-x_{m}\text{in}}. \end{array}$$

### 2.8. Analysis by DTW

The previous steps are framed in the algorithm within the adjustment of the signal and determining the cycles of the sample. Thus, returning to the concept of time series, an analysis is carried out in three aspects; from Pearson's iterations, the cyclical and seasonal component of the signal is determined, with the filter applied to the signals, with the noise being reduced, and finally with the normalization; it is intended to contribute to simplifying the comparison between samples by handling the same scale between them.

The DTW analysis is intended to make a comparison properly; this is done by determining the smallest distance between the two samples and its approximation to a line of slope one.

To define this algorithm process, we define two vectors *v*=[*x*_1_, *x*_2_, *x*_3_, *x*_4_,…, *x*_*n*_] and *u*=[*y*_1_, *y*_2_, *y*_3_, *y*_4_,…, *y*_*n*_], which have the same scale and are cyclical with the same number of samples per cycle.

If we review the distances between each sample of the two datasets, a distance matrix can be created, which can be seen in Table 1. For this case, it is defined as the cost matrix or distance matrix. In this case, there are high distances and small distances. When the distances are small, the samples are similar, and if the distances are high, the samples are distant.

**Figure 11:**
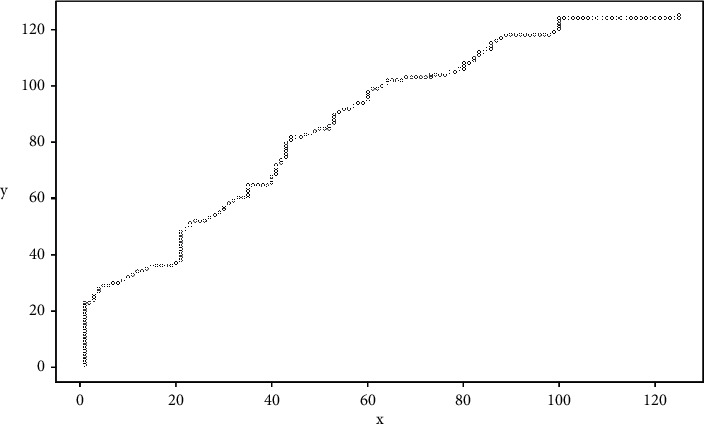
Example DTW mapping.

With the cost matrix, the most efficient path is defined; that is, it seeks to go through the matrix data where the cost is lower. Initial point is *c*=(*i*, *j*); you want to make a step *k* for another point *c*_*k*_=(*i*_*k*_, *j*_*k*_), in some adjacent side. Adjacent sides are (*x*_2_, *y*_1_), (*x*_2_, *y*_2_), and (*x*_1_, *y*_2_). The values for adjacent sides are (*x*_2_, *y*1)=(*y*_1_ − *x*_2_), (*x*_2_, *y*_2_)=(*y*_2_ − *x*_2_), and (*x*_1_, *y*_2_)=(*y*_2_ − *x*_1_). We search in the three adjacent sides the minimum value in such a way that the best path is recorded, considering that the lowest cost between the data represents the closeness between the data in the datasets, which corresponds to position *c*=(*i*, *j*), where the jump *c*_*k*_=(*i*_*k*_, *j*_*k*_) will be made towards the minimum value of these adjacent sides; that is, min((*y*_1_ − *x*_2_), (*y*_2_ − *x*_2_), (*y*_2_ − *x*_1_)). If we return to the example, with the initial data for vectors *v* and *u*, we can see the path mentioned in Figure 11.

**Figure 12:**
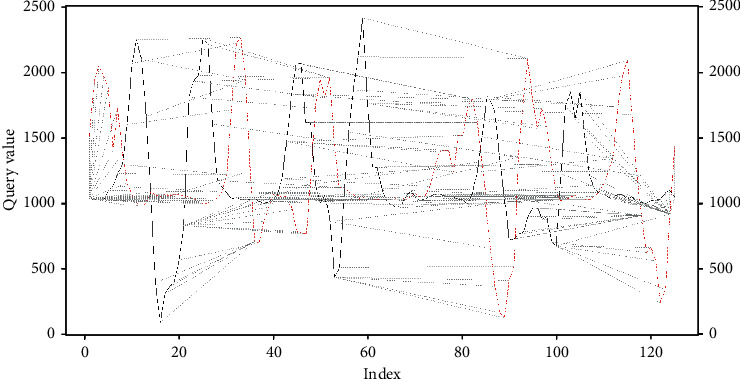
Example DTW signals.

In this sense, the approach between the two signals can be seen, where each line that joins the signals represents the optimal path found in the cost matrix; it is seen in Figure 12.

It must be taken into account that the examples shown in this section correspond to the signals without applying filters to them, without normalizing or adjusting, which is why the vertical and horizontal jumps are seen at the beginning and end of the route found by the cost matrix.

### 2.9. Comparative Analysis from DTW and Linear Regression

Starting from the cost matrix, we can consider that with greater approximation between two signals, taking into account the fact that the two signals are closer, the optimal path found from the cost matrix will tend to a secondary diagonal, which in case of representation would look similar to Figure 11.

**Figure 13:**
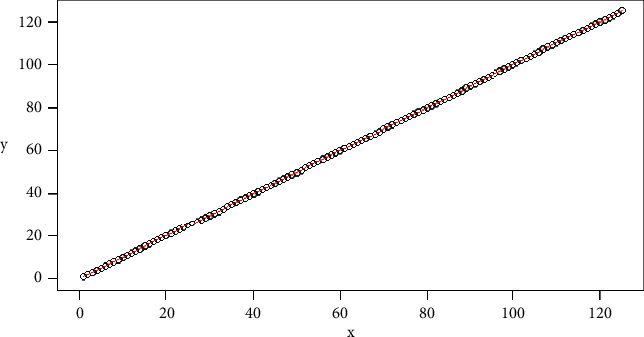
Example DTW-ideal signals.

An ideal case, where the values of a *v* signal are equal to the *u* values, both in the amount of data and in its behavior, the cost matrix will have a secondary diagonal with minimum values, and when mapping them in a plane, this will tend to a line *y*=*x*. Returning to the data assigned, we can do the DTW analysis for vector *v* on itself, and, as a result, for the first 124 pieces of data, we can see Figure 13. In this case, the line is overlapped with the mapping in the plane.

**Figure 14:**
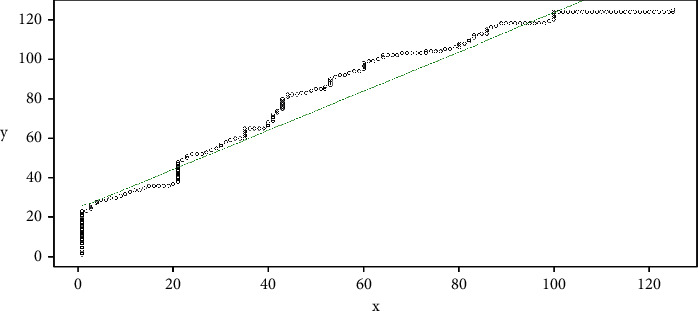
DTW and linear regression example.

From the mapping found for the optimal path, we can perform a linear regression to describe the behavior of the two signals. In this case, this is done using the least-squares method. Figures 11 and 13 can be associated from the statistics to scatter diagrams. From them, it can be seen with the naked eye that, in both cases, the data have an increasing behavior which can be summarized by drawing a line. With these characteristics, it is possible to find a line of the form *y*=mx+*b*, where *y* takes the role of the dependent variable and *x* that of the independent variable. In this case, *b* corresponds to the intercept with the *y*-axis, and *m* corresponds to the slope of the line. In the case of least-squares regression, the distance between the line and each of the dispersion points vertically should be the minimum possible. Thus, the distance between each dispersion point and the estimated line is known as the error. For the case of Figure 11 that comes out from *v* and *u* for the first 125 samples, the linear regression generated in Figure 14 can be observed. In this case, the line found obeys the equation *y*=0.9898*x*+24.4590, which is graphed with the green line.

In this way, we can conclude that, for two signals to obey a similar behavior, as long as the line generated from the linear regression of mapping the optimal is in a Cartesian plane, they approach the ideal path, which corresponds to the line *y*=*x*.

## 3. Results

### 3.1. Algorithm Developed

According to what is shown in Figure 2, the first step is the amount of data and to display it graphically.

- Data.Vector.1[ ] < −Expertdata
- Data.Vector.2[ ] < −Non − Expertdata
- Plot(Data.Vector1[ ], Data.Vector2[ ])

Then, the Pearson analysis is carried out for each signal and, as a result, the signal is obtained resulting from the Pearson iterations and the period of the signal; that is, every few data, there is a repetition of the movement and the first recommendation, where it is found if two samples have the same period. In this case, the result of the signals generated with the Pearson iterations is shown.

- Pearson Number Cicle1; Pearson Signal1[ ] < −Pearson Iteration(Data.Vecto1[ ])
- Pearson Number Cicle2; Pearson Signal2[ ] < −Pearson Iteration(Data.Vecto2[ ])
- Plot(Pearson Signal1[ ], Pearson Signal2[ ])
- if(Pearson Number Cicle1=Pearson Number Cicle2)
- {
- Cicle Equal < −Pearson Number Cicle1=Pearson Number Cicle2
- Continue⋯}
- Else {
- “The period of the samples is different”}

If the period of the samples is the same, the data is synchronized from the Pearson analysis shown in the “data adjustment” section of the two signals. On the other hand, we proceed that both signals start at the first maximum and that the two signals have the same number of elements.

- ⋯Continue
- Data.Vector1; Data.Vector2 < −Pearson Sync(Data.Vector1[ ], Data.Vector2[ ], Cicle Equal)
- Aux1[ ] < −Data.Vector1[1 : Cicle Equal]
- Aux2[ ] < −Data.Vector2[1 : Cicle Equal]
- Pos Max Aux1 < −Position where the maximum value is found for Aux1[ ]
- Pos Max Aux2 < −Position where the maximum value is found for Aux2[ ]
- Tam1 < −number of samples from Data.Vector1[ ]
- Tam2 < −number of samples from Data.Vector2[ ]
- Data.Vector1[ ] < −Data.Vector1[Pos Max Aux1 : Tam1]
- Data.Vector2[ ] < −Data.Vector2[Pos Max Aux2 : Tam2]
- if(Tam1 < Tam2)
- {
- Tam < −Tam1
- } else {
- Tam < −Tam2
- }
- Data.Vector1[ ] < −Data.Vector1[1 : Tam]
- Data.Vector2[ ] < −Data.Vector2[1 : Tam]

Once the signals are synchronized, and with the same number of samples, the signals are filtered to eliminate the noise, and the signals are normalized to adjust the data to the same scale according to the procedure shown in the sections “Filtering Data” and “Normalization.” The resulting signals are displayed at this point.

- Data.Vector.1[ ] < −Normalized Data(Data.Vector.1[ ])
- Data.Vector.2[ ] < −Normalized Data(Data.Vector.2[ ])
- Data.Vector.1[ ] < −Filter Data(Data.Vector.1[ ])
- Data.Vector.2[ ] < −Filter Data(Data.Vector.2[ ])
- Plot(Data.Vector1[ ], Data.Vector2[ ])

What has been done previously is due to the need to import and adjust the signals. Now, the comparison must be carried out as such, for which the DTW algorithm is used. In this case, according to the previous section, in “Analysis by DTW,” the path with the lowest values is obtained from a matrix of differences, which can be represented from a linear regression. In this case, the comparison function delivers the values of *m* and *b* to create the line representation (*y*=mx+*b*) to make the comparison. On the other hand, the value of “*R*-squared” determines if the values approximate the line to a greater or lesser extent, as well as its graphic representation.

It is essential to mention that what is described for the comparison can be done at both the beginning (for the original samples) and the end (for the adjusted samples).

Remember that when two signals are equal, the line corresponds to *y*=*x*. So we must determine how close the regression line found is to this one.

Our parameter that determines whether the movements made are similar occurs when the difference between each point that makes up our regression line, against the ideal line, tends to zero and when the *R*-squared tends to one.

It must be taken into account that the analysis must be carried out only with a sample of the entire signal generated. For this, we randomly determine carrying out the analysis of the signal only for one of the cycles of each sample.

- Sample Limit < −(Whole Part(Tam)/Cicle Equal) − 1
- Initial.Sample < −(Random Sample(1 : Sample Limit))*∗*Cicle Equal
- Final.Sample < −Initial.Sample+Cicle Equal1
- *m*; *b*; *R* − squared < −DTW Function(Data.Vector.1[Initial.Sample : Final.Sample];
- Data.Vector2[Initial.Sample : Final.Sample])
- *D* if[ ] < −Absolute value of the difference between the line*y* = *mx* + *b*and the liney=*x*
- for each point
- Diference Mesur < −mean(Absolute Value(*D* if))
- if(Diference Mesur =0ANDR − squared =1)
- {
- “The movement sare similar”
- } else{
- “the movement sare different”
- }
- Plot(straightlines, Difference of signals)

### 3.2. Case Study

A case study is carried out, showing the algorithm developed from a set of data captured with an accelerometer of a wearable device. The data captured corresponds to three salsa dance steps performed by three different people and, for each step, three samples are taken for each person. In this case, for each sample, about three thousand pieces of data are taken. For the three cases, the device was placed on the right ankle of each participant, and marks were made on the ground to limit the distance of the steps. The dance steps are “Step forward-backward,” “Step back-back,” and “Step side-side.” A 96 bpm metronome is used so that the steps are performed from a time mark. The data is in https://github.com/mfburbano/SalsaDanceDataSet. Capturing the dataset can be seen on these videos:

- Step forward-backward
- User 1: https://youtu.be/edGEdkCaSwM
- User 2: https://youtu.be/AStaTSjhMgU
- User 3: https://youtu.be/8bcM9MrA5Z8
- Step back-back
- User 1: https://youtu.be/KgBHLQuLmio
- User 2: https://youtu.be/BZz5Nm8zivs
- User 3: https://youtu.be/qyIjiD9l_5U
- Step side-side
- User 1: https://youtu.be/LRs7FhQZVeI
- User 2: https://youtu.be/tPPCZ3zAFD0
- User 3: https://youtu.be/gWcUhOwcflw

For this case, it is proposed that the difference between the regression lines is estimated at values lower than 10 and that the *R*-squared is more significant than 0.9. The comparisons that are given under these parameters will indicate the similarity in the movements.

From the dataset, the movements corresponding to User 1 will be taken as a sample of the expert and User 2 and User 3 as nonexperts.

The cases found when running the algorithm will be shown.

#### 3.2.1. Samples Are Similar

The data is obtained in the dataset of

(15) $$\begin{array}{c} \text{DatasetExpertSample}:\text{User}1\text{BackBackSample}1\\ \text{DatasetNon}-\text{Expert Sample}:\text{User}2\text{BackBackSample}3 \end{array}$$

The analysis by Pearson's iterations shows that the two samples have the same repetition period and that the samples are repeated every 124 pieces of data, therefore meeting the first criterion of similarity.

(16) $$\begin{array}{c} \text{PearsonNumberCicle}1=124\\ \text{PearsonNumberCicle}2=124 \end{array}$$

The random value, in this case, shows that the samples will be taken between Initial.Sample=372Final.Sample=496.

**Figure 15:**
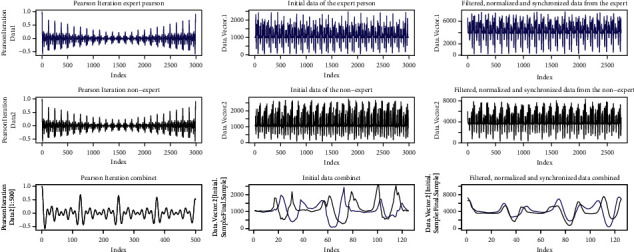
Signals similar movement.

Figure 15 shows the result of the Pearson iterations carried out in the left part. In the center, the figure shows the original signals, the expert person's signal is blue and the nonexpert signal is black. On the right, signals are shown after they have been synchronized, filtered, and normalized.

**Figure 16:**
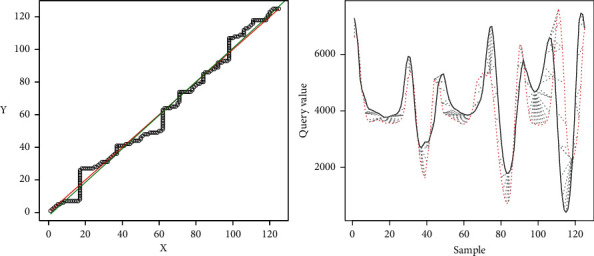
Results similar movement.

Figure 16 shows the line generated from the DTW analysis in green on the left side. The ideal line is shown in red and, in black, the coordinates are generated by the minor differences between the two samples. The right part shows the representation of the distances found for the two signals with the adjustment given by the DTW algorithm.

As a result, we have that *R* − Squared=0.9867Difference between line=0.903803.

In this case, the three conditions are met: the signals are periodic, and their period occurs every 124 samples. The *R*-squared is more significant than 0.9 and the difference between the lines is lower than 10. Therefore, it can be deduced that the movements are similar.

#### 3.2.2. Samples Are Different, *R*-squared < 0.9, and Regression < 10

The data is obtained in the dataset of

(17) $$\begin{array}{c} \text{DatasetExpertSample}:\text{User}1\text{BackBackSample}1\\ \text{DatasetNon}-\text{ExpertSample}:\text{User}2\text{BackBackSample}1. \end{array}$$

The analysis by Pearson's iterations shows that the two signals are repeated every 124 pieces of data; therefore, the first criterion of similarity is fulfilled.

(18) $$\begin{array}{c} \text{PearsonNumberCicle}1=124\\ \text{PearsonNumberCicle}2=124. \end{array}$$

The random value, in this case, shows that the samples will be taken between Initial.Sample=744Final.Sample=868.

**Figure 17:**
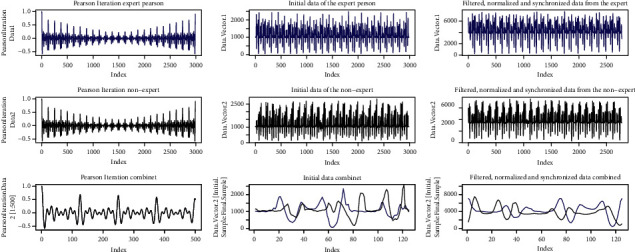
Signals, *R*-squared < 0.9; Regression < 10.

Figure 17 shows the signals of the Pearson iterations, the initial signals, and the signals after processing.

**Figure 18:**
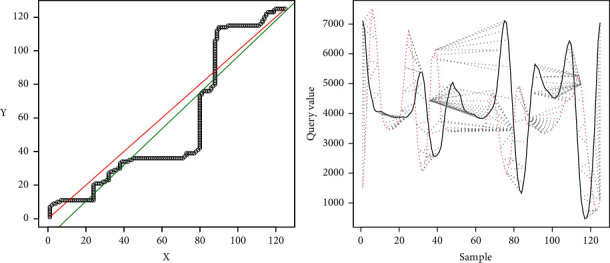
Results, *R*-squared < 0.9; Regression < 10.

Figure 18 shows the representation of the result of the comparison.

As a result, we have *R* − Squared=0.8482Difference between line=6.355008.

Since they do not meet the defined criteria, in this case, the transactions are different.

#### 3.2.3. Samples Are Different, *R*-squared > 0.9, and Regression > 10

The data are obtained in the dataset of

(19) $$\begin{array}{c} \text{DatasetExpertSample}:\text{User}1\text{Forward Backward Sample}1\\ \text{DataseNon}-\text{ExpertSample}:\text{User}3\text{Forward Backward Sample}2. \end{array}$$

The analysis by Pearson's iterations shows that the two signals are repeated every 125 pieces of data, thus meeting the first similarity criterion.

(20) $$\begin{array}{c} \text{PearsonNumberCicle}1=125\\ \text{PearsonNumberCicle}2=125. \end{array}$$

The random value in this case means that the samples will be taken between

(21) $$\begin{array}{c} \text{Initial}.\text{Sample}=1625\\ \text{Final}.\text{Sample}=1750. \end{array}$$

**Figure 19:**
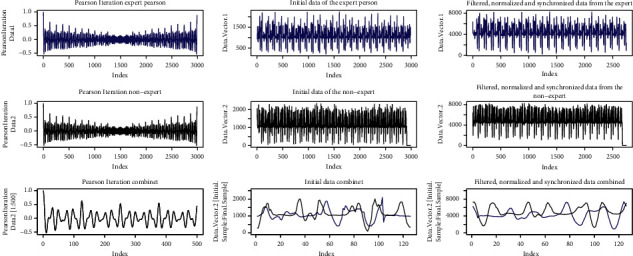
Signals, *R*-squared > 0.9; Regression > 10.

Figure 19 shows the signals from the Pearson iterations, the initial signals, and the signals after processing.

**Figure 20:**
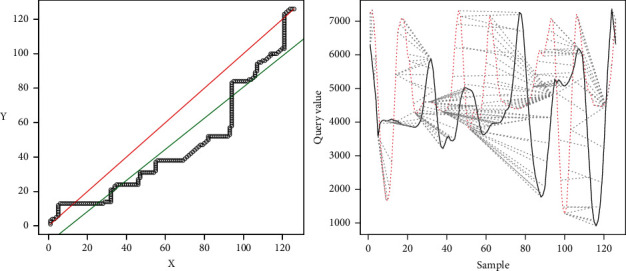
Results, *R*-squared > 0.9; Regression > 10.

Figure 20 shows the representation of the comparison result.

As a result, we have *R* − Squared=0.9099Difference between line=15.80045.

Since they do not meet the defined criteria, in this case, the movements are different.

#### 3.2.4. Samples Are Different Because They Have Different Periods

The data are obtained in the dataset of

(22) $$\begin{array}{c} \text{Dataset Expert Sample}:\text{User}1\text{Forward Backward Sample}3\\ \text{Dataset Non}-\text{Expert Sample}:\text{User}2\text{Forward Backward Sample}2. \end{array}$$

The analysis by Pearson iterations shows that the two signals have different periods.

(23) $$\begin{array}{c} \text{PearsonNumberCicle}1=13\\ \text{PearsonNumberCicle}2=124. \end{array}$$

**Figure 21:**
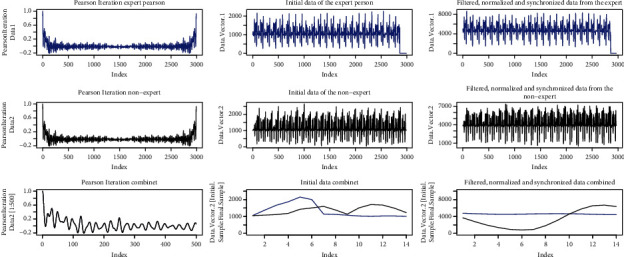
Signals, *R*-squared > 0.9; Regression > 10.

Figure 21 shows the signals from the Pearson iterations, the initial signals, and the signals after processing.

**Figure 22:**
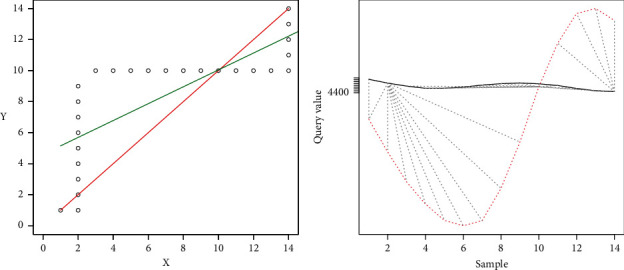
Results, *R*-squared > 0.9; Regression > 10.

Figure 22 shows the representation of the results of the comparison.

As a result, we have

(24) $$\begin{array}{c} R-\text{Squared}=0.5407\\ \text{ Differencebetweenline}=2.126166 \end{array}$$

Since they do not meet the criterion of equal periods, the movements are different.

## 4. Discussion

In the first case, where “the samples are similar,” in Figure 15, which maps the minor differences from DTW, it is observed that this mapping tends to the ideal straight line; therefore, the linear regression also tends to this ideal straight line; hence, *m* tends to 1 and *m* tends to 0. The *R*-squared tends to 1, so it is observed that the differences do not move away from the linear regression. Figure 16 corroborates that the two signals are pretty close to each other, and therefore the movements are similar.

The second case shows where the samples are different because *R*-squared *<* 0.9 and the difference of lines <10. It can be observed that the line generated from the linear regression is close to the ideal line, but the *R*-squared is far from 1, which implies that the data are scattered concerning the regression line. This dispersion implies that the algorithm makes jumps over the same point to achieve an optimal value, which leads to high values. Figure 18, on the right side, shows the above mentioned, where a high distance between the two signals is observed. This spacing shows that the correspondence occurs at different times for each signal between peaks and valleys, which can be translated as the movements being made at different times. In the particular case shown, it can be observed that the signals differ in the center, mainly, which would indicate that, at these moments, the movement is distorted. Meanwhile, on the extreme left, up to near the value of 50, the signals are pretty close. On the far right, near the value of 100 to 124, the signals again get closer to each other. The reading would imply that the user should correct the movements in the middle of the activity as they start and end correctly.

In the third case, the samples are different with *R*-squared >0.9, and the difference between the ideal line and the regression line is > 10. In this case, since the difference between the two lines is high, it is implied that the linear regression does not represent the movement of the expert, and therefore the movements are different. This is corroborated in Figure 20, on the left side, where it is observed that the two signals do not correspond. In this case, the user should improve the motion.

In the fourth case, the samples have different periods; therefore, the movements are not similar, shown in Figures 21 and 22. Here, Pearson's iterations are not generated in a regular way, as in the previous cases. The signals do not match either. In this case, the user should first try to have the same periodicity in the movements as those observed in the expert person.

## 5. Conclusions

The data captured and collected in the dataset used in this work can be treated as a time series for comparison. This requires the use of specific time series characteristics. Since we are dealing with data captured with periodic movements, the signals must have periodic characteristics. Thus, Pearson's iterations are used to deduce the values of the cyclic component and, through the use of filters, the noise components are eliminated.

Pearson's iterations can determine the periodicity of the signals. The comparison of this periodicity determines whether the person who is learning a movement performs it at the same speed and with the same periodicity as the experts. If the iterations give a different value for the two signals, the person who is learning is performing more or less minor movements than those indicated simultaneously or the speed at which he/she is doing them is higher or lower.

It is necessary to look for a process that allows synchronizing the two signals so that they are adjusted and the comparison can be made; therefore, the Pearson iterations are used again and, additionally, it is sought that the two signals start at a maximum value so that visually it is possible to identify if the synchronization is coincident.

The aim is to select only one sample for comparison; in this case, it is expected that the values of a particular cycle can be compared, so a function was developed in the algorithm to determine a particular cycle randomly for the two samples and to compare the values for this cycle. This was done because when the comparative analysis is performed for many values, the regression line always tended to the ideal line; likewise, the dispersion when many values were analyzed also tended to be low, which prevents a comparative analysis. However, if only one cycle is compared concerning another, it is possible to find particular values for comparison.

According to the smallest values in the distances in a Cartesian plane, the use of DTW features and their mapping of the ideal path is one of the most relevant aspects in the comparison performed. For this purpose, linear regression is used as a tool to assign comparable values. In this case, we look for the approximation to the ideal line of comparison and the dispersion of the data is not too large to determine the similarity between the samples. Thus, we use the average absolute value of the difference between the regression line and the ideal point-to-point line, and it is expected that difference will be small in similar movements. It is also expected that the data of the Cartesian plane do not disperse concerning the regression line. This can be seen in the value obtained from the linear regression with the *R*-squared. This value tends to 1 when the data are not very dispersed and tends to 0 when the values are dispersed, and the regression line does not represent the behavior of the values.

The graphics generated (Figures 16, 18, 20, and 22) represent, on the left side, the regression lines in green, the ideal lines in red, and the mapping of the ideal path from the DTW analysis. Moreover, on the right, the signal of the expert person is shown in black and that of the nonexpert in red. In this case, the differences are observed directly in the signal. If there is a sample with a partial similarity (as in Figure 18), it is possible to see these moments, where the similarity can be visually seen where the lines are close and the mapping is close to these two lines.

Finally, it is expected that this algorithm can be used in other datasets and other types of periodic motions. A real-time or approximate analysis, based on the algorithm and a similar analysis, but involving more variables in addition to acceleration is intended to be performed in the future.

## Figures and Tables

**Table 1:** Cost matrix.

*y*_*n*_	*y*_*n*_ − *x*_1_	*y*_*n*_ − *x*_2_	*y*_*n*_ − *x*_3_	*y*_*n*_ − *x*_4_	…	*y*_*n*_ − *x*_(*n* − 2)_	*y*_*n*_ − *x*_(*n* − 1)_	*y*_*n*_ − *x*_*n*_
*y*_(*n* − 1)_	*y*_(*n* − 1)_ − *x*_1_	*y*_(*n* − 1)_ − *x*_2_	*y*_(*n* − 1)_ − *x*_3_	*y*_(*n* − 1)_ − *x*_4_	…	*y*_(*n* − 1)_ − *x*_(*n* − 2)_	*y*_(*n* − 1)_ − *x*_(*n* − 1)_	*y*_(*n* − 1)_ − *x*_*n*_
*y*_(*n* − 2)_	*y*_(*n* − 2)_ − *x*_1_	*y*_(*n* − 2)_ − *x*_2_	*y*_(*n* − 2)_ − *x*_3_	*y*_(*n* − 2)_ − *x*_4_	…	*y*_(*n* − 2)_ − *x*_(*n* − 2)_	*y*_(*n* − 2)_ − *x*_(*n* − 1)_	*y*_(*n* − 2)_ − *x*_*n*_
⋮	⋮	⋮	⋮	⋮	⋮	⋮	⋮	⋮
*y*_4_	*y*_4_ − *x*_1_	*y*_4_ − *x*_2_	*y*_4_ − *x*_3_	*y*_4_ − *x*_4_	…	*y*_4_ − *x*_(*n* − 2)_	*y*_4_ − *x*_(*n* − 1)_	*y*_4_ − *x*_*n*_
*y*_3_	*y*_3_ − *x*_1_	*y*_3_ − *x*_2_	*y*_3_ − *x*_3_	*y*_3_ − *x*_4_	…	*y*_3_ − *x*_(*n* − 2)_	*y*_3_ − *x*_(*n* − 1)_	*y*_3_ − *x*_*n*_
*y*_2_	*y*_2_ − *x*_1_	*y*_2_ − *x*_2_	*y*_2_ − *x*_3_	*y*_2_ − *x*_4_	…	*y*_2_ − *x*_(*n* − 2)_	*y*_2_ − *x*_(*n* − 1)_	*y*_2_ − *x*_*n*_
*y*_1_	*y*_1_ − *x*_1_	*y*_1_ − *x*_2_	*y*_1_ − *x*_3_	*y*_1_ − *x*_4_	…	*y*_1_ − *x*_(*n* − 2)_	*y*_1_ − *x*_(*n* − 1)_	*y*_1_ − *x*_*n*_
	*x*_1_	*x*_2_	*x*_3_	*x*_4_	…	*x*_(*n* − 2)_	*x*_(*n* − 1)_	*x*_*n*_

## Data Availability

The corresponding dataset on salsa dance steps is available at https://github.com/mfburbano/SalsaDanceDataSet. The data can be used with referring to this paper. The data come from the capture through an inertial acceleration measure sensor placed on the ankle of the participants (captured at a rate of 96 bpm according to the videos linked in the document).
